# Comparing DNA methylation profiles in saliva and intestinal mucosa

**DOI:** 10.1186/s12864-019-5553-0

**Published:** 2019-02-28

**Authors:** Nerissa L. Hearn, Aaron S. Coleman, Vincent Ho, Christine L. Chiu, Joanne M. Lind

**Affiliations:** 1 School of Medicine, Western Sydney University, Sydney, Australia; 2 Faculty of Medicine and Health Sciences, Macquarie University, Sydney, Australia

**Keywords:** DNA methylation, Intestinal mucosal, Saliva

## Abstract

**Background:**

Altered epigenetic profiles are a feature of intestinal diseases, including ulcerative colitis and Crohn’s disease. DNA methylation studies in these diseases have utilised intestinal mucosal tissue or blood which can be difficult to collect, particularly for large-scale research studies. Saliva is an attractive alternative for epigenetic studies as it is easy to collect and provides high quality methylation profiles. The aim of the study was to determine the utility of saliva as an alternative for DNA methylation studies of intestinal disorders.

**Results:**

DNA methylation in saliva and intestinal mucosa samples were compared in individuals (*n* = 10) undergoing endoscopies using the Illumina Infinium Methylation 450 K Beadchip array. We found that DNA methylation was correlated between tissue types within an individual (Pearson correlation co-efficient r = 0.92 to 0.95, *p* < 0.001). Of the 48,541 probes (approximately 11% of CpG sites) that were differentially methylated between saliva and intestinal mucosa (adjusted p < 0.001, |Δβ| ≥ 20%), these mapped to genes involved in tissue-specific pathways, including the apelin signalling and oxytocin pathways which are important in gastrointestinal cytoprotection and motility.

**Conclusions:**

This study suggests that saliva has the potential to be used as an alternate DNA source to invasive intestinal mucosa for DNA methylation research into intestinal conditions.

**Electronic supplementary material:**

The online version of this article (10.1186/s12864-019-5553-0) contains supplementary material, which is available to authorized users.

## Background

Crohn’s disease, ulcerative colitis, coeliac disease, and irritable bowel disease are chronic intestinal inflammatory disorders characterised by varying intestinal symptoms including; severe diarrhoea, abdominal pain and rectal bleeding; as well as extra-intestinal complications including anaemia and malnutrition [1]. The development of these conditions is thought to be a result of a genetic predisposition coupled with environmental stimuli [2]. The involvement of both genetic and environmental factors suggests that epigenetic mechanisms have a role in the pathogenesis of these intestinal inflammatory diseases [3]. Epigenetic modifications are defined as stable and heritable alterations in gene expression and function that do not alter the DNA sequence [4]. DNA methylation is a type of epigenetic modification which regulates gene transcription and has roles in development, differentiation, and genomic stability [5]. It involves the addition of a methyl group to a cytosine nucleotide when it is followed by a guanine which are referred to as CpG dinucleotides. In the mammalian genome approximately 15% of the CpG dinucleotides are found in CpG islands, which are defined as stretches of DNA > 200 bp, with a CG content greater than 50% and an observed to expected ratio of CpG greater than 60% [6, 7]. CpG islands are often found in the promoter regions of both constitutively expressed genes and tissue specific genes [8]. Methylation of CpG islands are generally associated with gene repression.

Epigenetic modifications can be influenced by environmental factors including diet, illness and smoking [9]. DNA methylation has been implicated in the pathogenesis of intestinal inflammatory diseases, with studies in Crohn’s disease, inflammatory bowel disease, and ulcerative colitis demonstrating that individuals with these conditions have altered DNA methylation profiles in their intestinal, colon, or rectal tissue, when compared to healthy controls [2, 10, 11].

Many studies have investigated the utility of DNA methylation biomarkers for disease screening, diagnosis, and management [12, 13]. Epigenetic studies in inflammatory intestinal diseases have utilised either intestinal mucosal tissue or peripheral blood mononuclear cells [14, 15]. Collection of these samples is invasive, carries the risk of complications, and requires trained personnel, which for large-scale research studies can be problematic. Saliva is an attractive alternative for epigenetic studies as it is easy to collect and provides high quality methylation profiles [16]. In the clinical setting, saliva is also an attractive alternative compared to blood or intestinal mucosa, as individuals can collect their own saliva using commercially available kits which stabilises the sample at room temperature, enabling individuals to mail the sample back to the clinic. This negates the need for specially trained medical personnel and reduces the risk of disease transmission via needle stick injury [17]. Furthermore, the convenience of this method may also increase compliance rates for follow up medical appointments and procedures [16]. Studies examining the suitability of saliva as a source of DNA for epigenetic and genetic research have shown that saliva yields DNA of sufficient quantity and quality for downstream applications [18].

DNA methylation studies comparing profiles between tissue types within individuals have shown that regions of tissue-specific differential methylation mainly map to CpG poor regions [19]. The viability of saliva as an alternative for less accessible tissues; including brain [20], lung/bronchial epithelium [21], and peripheral blood mononuclear cells [16], for DNA methylation studies has been previously demonstrated, with methylation profiles correlating positively between saliva and the tissue in question. Saliva is composed of more than 99% water, and also contains white blood cells and epithelial cells, which represent the cell types of the oral mucosa [22]. The oral cavity is connected to and represents the entrance to the gastrointestinal tract. For studies where environmental exposures can drive epigenetic change, saliva and the oral mucosa is an ideal alternative for the intestinal mucosa given the similarities in cellular composition and environmental exposures between the two tissue sites.

To date, no studies have compared DNA methylation profiles between saliva and the intestinal mucosa. The aim of the current study was to determine the utility of saliva for large scale DNA methylation research studies in intestinal conditions by investigating tissue-specific methylation differences.

## Results

Intestinal mucosa and saliva samples were obtained from 10 individuals. All individuals were female, Caucasian, and were not current smokers. The mean age was 51.6 ± 6.1 years old, and the mean BMI was 28.5 ± 1.8 kg/m^2^. Individuals were undergoing endoscopies for investigations into epigastric pain and difficulty swallowing or were having routine follow up endoscopies for coeliac disease. All individuals with coeliac disease were currently on a gluten-free diet and did not have active disease. Demographic and clinical characteristics of the participants are summarised in Table 1.

**Table 1:** Clinical and demographical characteristics of individuals

Patient	Age range	BMI	Ethnicity	Smoking Status	Reason for endoscopy	Medications
One	65–74	30.0	Caucasian	Quit	Difficulty swallowing	Somac, Warfarin
Two	65–74	33.6	Caucasian	Never	Epigastric pain	
Three	65–74	39.9	Caucasian	Never	Epigastric pain	Nexium
Four	45–54	28.4	Caucasian	Quit	Oesophageal reflux	Somac
Five	18–25	25.4	Caucasian	Never	Oesophageal reflux	Nexium, Motilium, Mezavart
Six	45–54	30.1	Caucasian	Never	Routine follow up for coeliac disease	
Seven	35–44	26.3	Caucasian	Quit	Routine follow up for coeliac disease	
Eight	55–64	24.3	Caucasian	Never	Routine follow up for coeliac disease	
Nine	65–74	29.0	Caucasian	Quit	Routine follow up for coeliac disease	
Ten	18–25	17.8	Caucasian	Never	Routine follow up for coeliac disease	

**Fig. 1:**
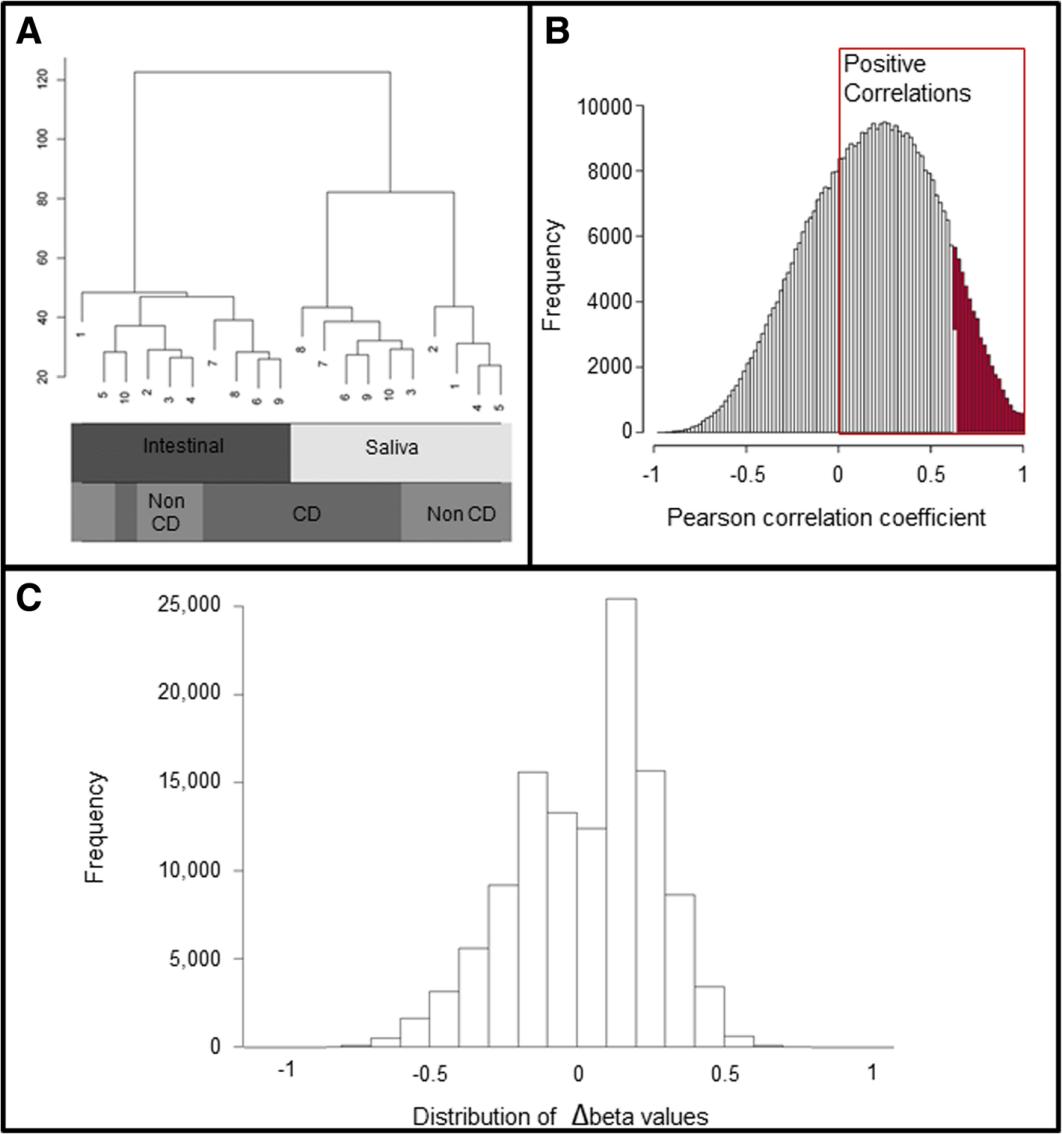
Data clustering and comparison between saliva and tissue DNA methylation profiles. **a**) Hierarchical clustering based on correlation distances of methylation profiles in 20 samples – intestinal mucosa and saliva samples from 10 individuals; **b**) Pearson correlation coefficients for methylation levels of saliva vs tissue for each of the CpG sites (positive correlations in boxed area with statistically significant correlations (adjusted *P* < 0.05) shown in red bars); **c**) The distribution of cg-probes plotted against the difference in methylation expressed as Δβ-values

**Fig. 2:**
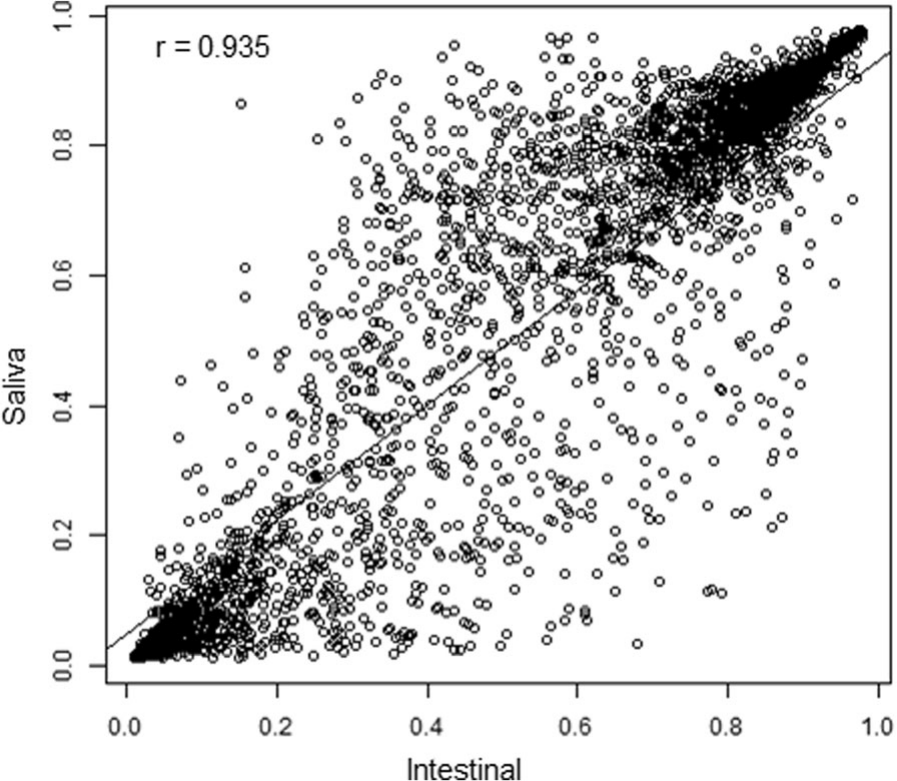
Scatter plot of average DNA methylation beta values for 4000 random CpG sites between intestinal and saliva samples. Line represents the line of best fit

DNA methylation was measured using Illumina Infinium Methylation 450 K Beadchip array for all 20 samples (10 saliva and 10 matched intestinal samples). Following quality control filtering and removal of probes on sex chromosomes and probes on non-cpg sites, 456,148 probes were included in the analysis. Hierarchical clustering based on average linkage and correlation-based distances showed that the saliva and intestinal tissue samples clustered into two distinct groups, and within the saliva cluster, individuals with coeliac disease also appear to cluster together (Fig. 1a). Similar patterns were observed following separation of significant and non-significant probes (Additional file 1: Figure S2). Multidimensional scaling (MDS) of the top 1000 most variable sites showed intestinal mucosal tissue clustered tightly, while saliva samples were more variable (Additional file 2: Figure S1C). Overall, global DNA methylation of saliva and intestinal mucosa was positively correlated within an individual (Pearson’s correlation co-efficient r 0.92–0.95, *p* < 0.001) (Table 2). For 68.9% of CpG sites positive Pearson correlations between methylation levels of saliva and tissue samples were observed, and of these 14.8% were statistically significant (Fig. 1b). A scatter plot of average DNA methylation values for 4000 random CpG sites indicates methylation is in the same direction in saliva versus intestinal mucosal tissue samples, r = 0.94 (Fig. 2). The distribution of difference in DNA methylation (|Δβ|) between saliva and tissue samples is shown in Fig. 1c. Using the filtering criterion of adjusted *p <* 0.001, |Δβ| ≥20%, 48,541 probes were identified as being differentially methylated, of which 20,152 (41.5%) were hypomethylated and 28,389 (58.5%) hypermethylated in saliva vs intestinal mucosa (Additional file 3: Table S1). Using both an effect size and the *P* value cut-off (adjusted *p* < 0.001, mean |Δβ| ≥20%), region analysis identified 292 tissue-specific regions (tDMR), 21 (7.2%) hypomethylated and 271 (92.8%) hypermethylated regions between saliva and intestinal mucosa, which mapped to 278 annotated genes (Additional file 4: Table S2).

**Table 2:** Correlation of average DNA methylation across all CpG sites between saliva and intestinal mucosa within an individual

Participant	r-value	*p*-value	Confidence Interval
One	0.927	*p* < 0.001	0.926–0.927
Two	0.950	*p* < 0.001	0.950–0.951
Three	0.921	*p* < 0.001	0.920–0.921
Four	0.939	*p* < 0.001	0.938–0.939
Five	0.947	*p* < 0.001	0.946–0.947
Six	0.928	*p* < 0.001	0.928–0.929
Seven	0.922	*p* < 0.001	0.922–0.923
Eight	0.924	*p* < 0.001	0.923–0.924
Nine	0.930	*p* < 0.001	0.930–0.930
Ten	0.935	*p* < 0.001	0.935–0.936

Functional annotation analysis of the 278 annotated genes identified cell adhesion (GO:0098609, GO:0007156, GO:0098742, GO:0007155), biological adhesion (GO:0022610) and calcium ion binding (GO:0005509) as the top most significant terms (Table 3). Pathway analysis identified apelin signalling pathway, regulation of actin cytoskeleton, transcriptional mis-regulation in cancer, oxytocin signalling and metabolic pathways as being significantly enriched pathways in these regions (Table 4).

**Table 3:** GO terms for differentially methylated genes between intestinal mucosa and saliva samples

GO No.	GO Term	Count	*P* value	Benjamini *P* values
GO:0007156	homophilic cell adhesion via plasma membrane adhesion molecules	23/154	*p* < 0.001	*p* < 0.001
GO:0098742	cell-cell adhesion via plasma-membrane adhesion molecules	24/221	*p* < 0.001	*p* < 0.001
GO:0005509	calcium ion binding	33/684	*p* < 0.001	*p* < 0.001
GO:0098609	cell-cell adhesion	43/1124	*p* < 0.001	*p* < 0.01
GO:0007155	cell adhesion	53/1656	*p* < 0.001	*p* < 0.01
GO:0022610	biological adhesion	53/1662	*p* < 0.001	*p* < 0.01

*P* value based on number of differentially expressed genes

Count is the number of genes within the test set over the total of number of genes within the GO term

**Table 4:** Pathway analysis for differentially methylated genes between intestinal mucosa and saliva samples

	Pathway	Count	*P* value	Benjamini *P* values
hsa04371	Apelin signalling pathway	5/137	*p* < 0.001	0.01
hsa04810	Regulation of actin cytoskeleton	6/211	*p* < 0.001	0.01
hsa05202	Transcriptional misregulation in cancer	6/174	*p* < 0.001	0.01
hsa01100	Metabolic pathways	11/1241	*p* < 0.001	0.01
hsa04022	cGMP-PKG signalling pathway	5/162	*p* < 0.001	0.01
hsa04070	Phosphatidylinositol signalling system	4/97	*p* < 0.001	0.01
hsa04144	Endocytosis	6/257	*p* < 0.001	0.01
hsa04666	Fc gamma R-mediated phagocytosis	4/90	*p* < 0.001	0.01
hsa04621	NOD-like receptor signalling pathway	4/158	*p* < 0.001	0.01
hsa04020	Calcium signalling pathway	5/180	*p* < 0.001	0.01
hsa04915	Estrogen signalling pathway	4/98	*p* < 0.001	0.02
hsa04724	Glutamatergic synapse	4/114	*p* = 0.001	0.03
hsa04662	B cell receptor signalling pathway	3/70	*p* = 0.001	0.03
hsa05132	Salmonella infection	3/85	*p* = 0.001	0.03
hsa04062	Chemokine signalling pathway	4/182	*p* = 0.002	0.04
hsa04921	Oxytocin signalling pathway	4/152	*p* = 0.002	0.04

*P* value based on number of differentially expressed genes

Count is the number of genes within the test set over the total of number of genes within the GO term

## Discussion

The present study compared DNA methylation profiles between saliva and intestinal mucosa within an individual, to determine the feasibility of saliva as an alternative for intestinal mucosal tissue for DNA methylation research studies in intestinal conditions. We found that methylation profiles were highly correlated between saliva and intestinal mucosa (r 0.92–0.95) within an individual. This indicates that saliva has the potential to be a viable alternative for intestinal tissue for future DNA methylation studies.

Saliva is an attractive alternative for DNA methylation profiling as collection is simple, non-invasive, and high quality methylation profiles can be generated [16, 18]. For participant recruitment in large research studies, these factors are highly beneficial for increasing participation as seen in a study comparing participation between blood collection (31% positive) and saliva collection (72% positive) [23]. This could be extended to a clinical setting where the potential use of saliva to help screen and monitor conditions, may improve compliance rates for patient follow up appointments.

The feasibility of using oral mucosa as a potential alternative for a less accessible tissue has been previously investigated. Methylation profiles comparing buccal epithelial swabs and bronchial epithelium obtained from individuals with adenocarcinoma lung cancers who smoke tobacco, to assess the utility of saliva for screening and diagnosing lung cancer found the methylation profiles of two genes involved in early lung carcinogenesis, *p16* and *FHIT*, correlated strongly between saliva and bronchial samples [21]. It was suggested that since the entire airway from oral cavity to lungs is exposed to the carcinogens from tobacco, the carcinogens would be able to induce similar epigenetic alterations in the oral mucosa as well as in the distant lung tissue. This study was the first to suggest that an environmental agent could induce the same epigenetic changes in the oral mucosa as it does in a distant tissue, prompting the suggestion that oral mucosa could be used as a surrogate tissue in early screening interventions for preventing lung cancer [21]. In respiratory allergy, comparisons between peripheral blood mononuclear cells and saliva found that 96% of CpG sites were comparable between peripheral blood mononuclear cells and saliva [16]. Further comparisons between saliva and blood in a population of African Americans recruited at urban public hospitals found 88.6% of CpG sites were comparable. This study also found that DNA methylation profiles between saliva and brain tissues were more similar than methylation profiles between saliva and blood [20].

The oral cavity is the entrance to the gastrointestinal tract. The gastrointestinal tract is a muscular tube lined by a mucous membrane with similar histological organization across its segments; the oral cavity, stomach, small intestine and large intestine. Oral mucosa consists of two layers, the surface stratified squamous epithelium and the deeper lamina propria. Duodenal mucosa consists of three layers, columnar epithelium with absorptive capacity including villi, lamina propria and a deep submucosa lined with Brunner’s glands which secrete mucus and bicarbonate in order to neutralise stomach acids and is responsible for the enzymatic breakdown of food. Enzymes found in saliva are also essential in the breakdown of dietary starches and fats. The similar composition and function of mucosa between the oral mucosa and intestinal mucosa alongside our findings of comparable methylation profiles between saliva and intestinal mucosa strengthens the idea that saliva has the potential to be used as an alternative for more invasive tissues.

When comparing tissue types within an individual, a proportion of CpG sites will be differentially methylated, due to tissue-specific functions, which can be seen in Additional file 5: Figure S3. Studies comparing methylation profiles between tissue types within individuals have identified tissue-specific differentially methylated regions (tDMRs) using the criterion absolute methylation differences > 20% and *p*-value < 0.001 [16, 19, 24, 25]. Methylation comparisons between 17 different human somatic tissues, including gastric mucosa found that 2.2% of hypermethylated and 14.9% of hypomethylated CpG sites were commonly seen across all 17 tissue types. Comparisons between a specific tissue type and all other tissue types identified 14,441 tDMRs. Functional analysis of the tDMR found the genes that were hypomethylated in certain tissues were frequently associated with tissue-specific functions, while none of the hypermethylated genes were found to be involved in tissue-specific functions, indicating that hypomethylation is associated with tissue-specific functions [24].

In the present study, 292 tDMR were identified between intestinal mucosa and saliva which corresponded to 278 unique genes. Pathway analysis of these genes identified apelin signalling pathways as being significantly enriched. Apelin is an important gut regulatory peptide ligand of the APJ receptor which has a role in gastrointestinal cytoprotection by regulating apoptosis [26, 27]. The oxytocin signalling pathway was also significantly enriched. Oxytocin has been suggested to contribute to the control of the gastrointestinal motility, as it is expressed in nerve fibres along the entire human gastrointestinal tract, [28]. The differences in methylation profiles observed are expected when comparing between two different tissue types.

Samples were collected from individuals who were undergoing investigations for upper gastrointestinal related issues. Comparisons of methylation profiles were made within individuals which likely minimised any effect of the different gastric issues on the methylation profiles. Many upper gastric issues, for example gastro-oesophageal reflux affect both the upper gastric region and the oral cavity. Despite the main damage occurring to the lower oesophagus, oral manifestations of gastroesophageal reflux can include a burning mouth sensation, tongue sensitivity, itching/burning, tooth erosion, dentinal hypersensitivity and temporomandibular/myofascial pain dysfunction [29, 30]. Similarly, in coeliac disease where the typical symptoms are observed in the abdominal region, the disease may manifest in the oral cavity as oral ulcerations, changes in the salivary and parotid glands, and changes in the tongue [31].

The gut microbiome can effect DNA methylation profiles via the production of metabolites, which alters the pool of compounds used in epigenetics modifications, or through inhibiting enzymes involved in epigenetic pathways [32]. The differences in methylation profiles observed may be due to differences in the microbiota of these two tissues. Further investigations would be needed to assess if there are differences in the microbiome profiles between the oral and intestinal microbiota and if these are causing the differences observed in the methylation profiles.

In addition to the clustering of saliva and intestinal tissue into two distinct groups, saliva samples also clustered into two distinct groups. These two clusters appear to be the individuals with coeliac disease and individuals being investigated for other gastrointestinal disorders. All individuals with coeliac disease were currently on a strict gluten-free diet. Diet has been shown to modify microbiota populations as well as methylation profiles [33–35]. Differences in microbial populations due to diet, or the presence of coeliac disease, could account for the clustering observed. Further investigations comparing DNA methylation profiles from saliva samples between coeliac and non-coeliac individuals in a larger dataset would be of interest. Furthermore, the post normalisation MDS plots (Additional file 2: Figure S1C) shows tight clustering of intestinal samples but greater variations in saliva sample. This suggests that saliva is a potentially more informative tissue compared to intestinal mucosa with regard to DNA methylation due to the ability to detect differences between disease groups.

Half of our participants were former smokers, while smoking can have long-lasting effects on DNA methylation patterns, it has been shown that individuals who quit smoking have DNA methylation profiles similar to those of non-smokers [36]. It has been suggested however that environmental agents can induce the same epigenetic changes in the oral mucosa as it does in distant tissue [21]. Therefore, any methylation changes caused by past smoking in the oral mucosa could occur in the gut as well. Comparisons were conducted within an individual to account for the effects of past smoking on DNA methylation profiles.

## Conclusion

The results from this study provide a framework for the use of saliva in DNA methylation research studies in intestinal conditions. It suggests that saliva has the potential to be used as an alternative for intestinal mucosa which would be beneficial to epigenetic research in inflammatory intestinal conditions by negating the need for invasive tissue collection. Overall, positive correlations in global DNA methylation were observed between saliva and intestinal mucosa tissues. The presence of inter-individual variation in saliva, particularly the clustering of individuals with coeliac disease compared to non-coeliac disease individuals, is of particular interest for research studies that aim to identify epigenetic risk factors that are associated with disease. Further studies investigating the influence of a gluten-free diet on salivary DNA methylation profiles would be of interest.

## Methods

### Participant recruitment and sample collection

Individuals undergoing an endoscopy to investigate non-cancer related epigastric symptoms at Campbelltown Private Hospital, NSW, Australia were recruited into this study. This study was approved by the Western Sydney University Human Ethics Committee (H10513), the South Western Sydney Local Health District Ethics Committee (HREC/16/LPOOL/305) and the Macquarie University Human Ethics Committee (5201700199). Following written informed consent, demographic, clinical, and lifestyle information was collected via questionnaire [37]. Saliva samples were collected immediately prior to the patient’s endoscopy using the Oragene-DNA OG500 saliva self-collection kit (DNA Genotex, Canada), and stored at 4 °C. Intestinal biopsy samples were collected by the gastroenterologist during an upper gastric endoscopy procedure, while the participant was under sedation. Biopsies were excised from the duodenal region of the gastrointestinal tract and stored at − 80 °C.

### DNA extraction

Genomic DNA was extracted from intestinal mucosal tissue using the Qiagen DNA Mini Kit *Tissue Spin Column Protocol* (Qiagen, Germany). Saliva was incubated at 50 °C for 2 h followed by incubation with proteinase K at 56 °C for 10 min. Saliva samples were processed using the prepIT-L2P DNA collection kit (DNA Genotek, Canada) according to the manufacturer’s protocol. Saliva DNA was then purified using the Qiagen DNA mini kit spin column protocol (Qiagen, Germany). All extracted samples were quantified and stored at − 20 °C.

### Illumina Infinium HumanMethylation450 BeadChip analysis

Genomic DNA underwent sodium bisulfite conversion and was hybridized to the Illumina Infinium HumanMethylation450 BeadChip (Illumina, San Diego, CA). Amplification, hybridization, washing, labelling and scanning of the array was performed by the Australian Genome Research Facility (AGRF), a fee-for-service provider. Raw IDAT files containing signal intensities for each probe were extracted using Illumina GenomeStudio software and imported into *RStudio* using the *methylumi* and *minfi* packages [38, 39]. Data quality control and processing steps were conducted using the *methylumi* and *wateRmelon* packages in R [38, 40]. Filtering by detection *p*-values removed both failed samples and probes. The *pfilter* function was used to discard probes with a detection *p*-value > 0.01 in at least 1% samples (*n* = 1011) and/or a bead count less than 3 in 5% of samples (*n* = 240). No samples were removed. Data was normalised by adjusting for background methylated and unmethylated intensities with the *dasen* function [40]. Multi-dimensional scaling (MDS) plots of variably methylated probes in saliva and intestinal mucosa were used to confirm that the predicted tissue type matched the reported tissue type for each participant pre and post normalisation (Additional file 2: Figure S1).

Methylation measurements of a subset of probes on the microarray can be confounded by underlying SNPs and cross-hybridisation to other areas of the genome. To account for this, probes containing a SNP with minor allele frequency (MAF) > 5% within 5 bp of the single base extension site were removed from all analyses based on the SNP annotation data provided by Illumina, the Bioconductor package *minfi* [39], and Chen and colleagues [41]. Probes located on sex chromosomes (X chromosome *n* = 11,141 and Y chromosome *n* = 416), and non-cpg probes (*n* = 3073) were also removed from subsequent analysis, leaving 456,148 probes. Hierarchical clustering was performed using average linkage and correlation-based distances metric for clustering. Saliva contains a mixture of different cell types, and cell-type proportions may differ across individuals [42]. Surrogate variable analysis using the *sva* package was used to identify sources of variation, including cell type heterogeneity within samples and potential batch effects [43, 44]. *sva* using the “*leek*” method identified two surrogate variables that were then adjusted for in subsequent analysis.

Moderated paired t-tests were used to assess the association between methylation and tissue type at the individual probe level using the *limma* package [45]***.*** Prior to analysis, β-values were log transformed to M-values to improve sensitivity. *P*-values were adjusted for multiple testing according to the false discovery rate (FDR) procedure of Benjamini Hochberg. Significantly differentially methylated probes (DMPs) were selected using an absolute β difference of ≥20% and an adjusted *p* < 0.001 cut-off. While DNA methylation studies can report effect sizes as small as 5% [15], a more stringent definition of absolute beta difference of 20% was used as we would expect larger differences between tissue types. Furthermore, these cut-offs are in line with previous studies comparing DNA methylation between tissues [16, 19, 24, 25]. The *DMRcate* package [46] was then used to identify differentially methylated regions (DMRs), based on consecutive CpG probes with a mean methylation difference above a certain threshold. The inclusion threshold was set to beta difference ≥ 20%, and at least two consecutive CpGs to minimise the probability of randomly obtaining consecutive CpGs whose mean effect size are above 20%. Correlations between methylation in saliva and intestinal mucosa were measured using the *cor.test* function, at each probe site between paired samples, using Pearson’s product moment correlation coefficient. A Benjamini-Hochberg adjusted *p*-value < 0.05 was considered as significant.

### Gene ontology

Functional annotation analysis and gene ontology (GO) enrichment analysis was performed using the *missMethyl* package [47]. The *gometh* function (prior.prob. = TRUE) was used to test gene ontology enrichment for significant CpGs. In addition, the *gometh* function was used to perform pathway enrichment analysis based on the *Kyoto Encyclopedia of Genes and Genomes* (KEGG) classification databases to identify significant pathways. Following this, the *topGO* or *topKEGG* function of the *limma* package was used to identify the most significant GO terms and KEGG pathways.

## Additional files

Additional file 1: **Figure S2**. Hierarchical clustering. A. Only significant probes, and B. Only non-significant probes. Significance is based on absolute beta difference of 20% and *p* < 0.001. (PNG 39 kb)

Additional file 2: **Figure S1.** Multi-dimensional scaling. A. All samples pre-normalisation samples classified by tissue type. B. All samples pre-normalisation samples classified by whether individuals have coeliac disease. C. All samples post-normalisation samples classified by tissue type. D. All samples post-normalisation samples classified by whether individuals have coeliac disease. (PNG 40 kb)

Additional file 3: **Table S1.** Significantly differentially methylated probe sites between intestinal mucosa and saliva. (CSV 3408 kb)

Additional file 4: **Table S2.** Significantly differentially methylated regions between intestinal mucosa and saliva. (CSV 17 kb)

Additional file 5: **Figure S3.** Manhattan Plot. Manhattan plot of each CpG site plotted according to genomic position on the x-axis and the strength of the association (−log10 *p*-value) on the y-axis. The higher red line is the “Bonferroni” adjusted significance cut-off of –log10(5 × 10^− 8^), while the lower blue line is the “Candidate” cut-off of –log10(5 × 10^− 6^). (PNG 69 kb)

## Abbreviations

DMP: Differentially methylated probe
DMR: Differentially methylated region
FDR: False discovery rate
GO: Gene ontology
MAF: Minor allele frequency
tDMR: Tissue-specific differentially methylated region
