# Supplementary figures and images for: Comparing DNA methylation profiles in saliva and intestinal mucosa
Source: BMC Genomics. 2019 Feb 28;20:163. doi: 10.1186/s12864-019-5553-0 (PMC6394071; doi:10.1186/s12864-019-5553-0)

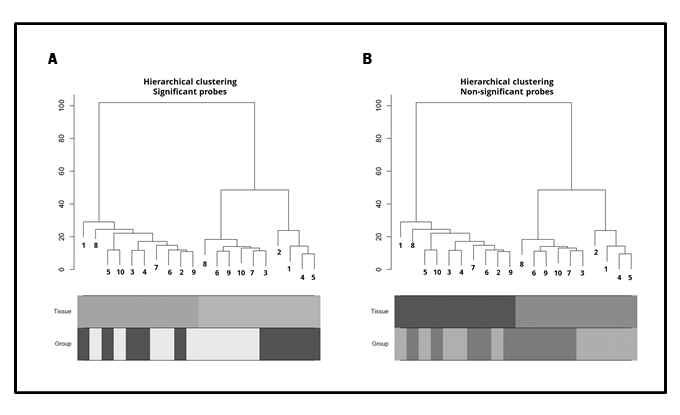

Supplement: Supplementary file 1 — Figure S2. Hierarchical clustering. A. Only significant probes, and B. Only non-significant probes. Significance is based on absolute beta difference of 20% and p < 0.001. (PNG 39 kb) [file 12864_2019_5553_MOESM1_ESM.png]

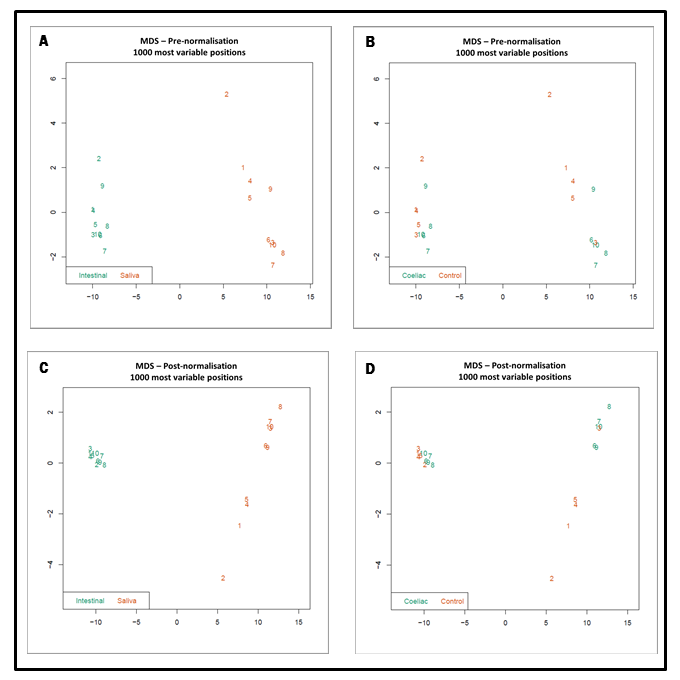

Supplement: Supplementary file 2 — Figure S1. Multi-dimensional scaling. A. All samples pre-normalisation samples classified by tissue type. B. All samples pre-normalisation samples classified by whether individuals have coeliac disease. C. All samples post-normalisation samples classified by tissue type. D. All samples post-normalisation samples classified by whether individuals have coeliac disease. (PNG 40 kb) [file 12864_2019_5553_MOESM2_ESM.png]

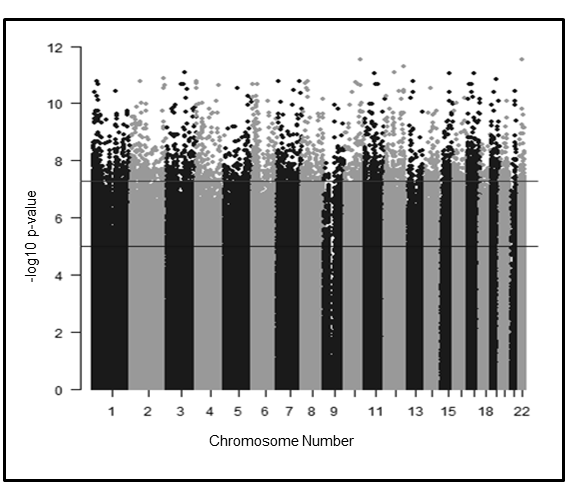

Supplement: Supplementary file 5 — Figure S3. Manhattan Plot. Manhattan plot of each CpG site plotted according to genomic position on the x-axis and the strength of the association (−log10 p-value) on the y-axis. The higher red line is the “Bonferroni” adjusted significance cut-off of –log10(5 × 10− 8), while the lower blue line is the “Candidate” cut-off of –log10(5 × 10− 6). (PNG 69 kb) [file 12864_2019_5553_MOESM5_ESM.png]
